# Pterostilbene Promotes Mean Lifespan in Both Male and Female *Drosophila Melanogaster* Modulating Different Proteins in the Two Sexes

**DOI:** 10.1155/2022/1744408

**Published:** 2022-02-16

**Authors:** Daniela Beghelli, Lorenzo Zallocco, Maria Cristina Barbalace, Simona Paglia, Silvia Strocchi, Ilenia Cirilli, Valeria Marzano, Lorenza Putignani, Giulio Lupidi, Silvana Hrelia, Laura Giusti, Cristina Angeloni

**Affiliations:** ^1^School of Biosciences and Veterinary Medicine, University of Camerino, Via Gentile III da Varano, 62032 Camerino (MC), Italy; ^2^Department of Pharmacy, University of Pisa, 56126 Pisa, Italy; ^3^Department for Life Quality Studies, Alma Mater Studiorum, University of Bologna, Corso d'Augusto 237, 47921 Rimini (RN), Italy; ^4^Department of Pharmacy and Biotechnology, University of Bologna, Via Selmi 3, 40126 Bologna (BO), Italy; ^5^Translational Research Lab-Santa Maria Nuova Hospital-IRCCS, Viale Umberto I 50, 42123 Reggio Emilia, Italy; ^6^School of Pharmacy, University of Camerino, Via Gentile III da Varano, 62032 Camerino (MC), Italy; ^7^Multimodal Laboratory Medicine Research Area, Unit of Human Microbiome, Bambino Gesù Children's Hospital, IRCCS, Viale di San Paolo 15, 00146 Rome, Italy; ^8^Department of Diagnostics and Laboratory Medicine, Unit of Microbiomics and Multimodal Laboratory Medicine Research Area, Unit of Human Microbiome, Bambino Gesù Children's Hospital, IRCCS, Viale di San Paolo 15, 00146 Rome, Italy

## Abstract

Aging is a multifactorial phenomenon characterized by degenerative processes closely connected to oxidative damage and chronic inflammation. Recently, many studies have shown that natural bioactive compounds are useful in delaying the aging process. In this work, we studied the effects of an *in vivo* supplementation of the stilbenoid pterostilbene on lifespan extension in *Drosophila melanogaster*. We found that the average lifespan of flies of both sexes was increased by pterostilbene supplementation with a higher effect in females. The expression of longevity related genes (Sir2, Foxo, and Notch) was increased in both sexes but with different patterns. Pterostilbene counteracted oxidative stress induced by ethanol and paraquat and up-regulated the antioxidant enzymes Ho e Trxr-1 in male but not in female flies. On the other hand, pterostilbene decreased the inflammatory mediators dome and egr only in female flies. Proteomic analysis revealed that pterostilbene modulates 113 proteins in male flies and only 9 in females. Only one of these proteins was modulated by pterostilbene in both sexes: vacuolar H[+] ATPase 68 kDa subunit 2 (Vha68-2) that was strongly down-regulated. These findings suggest a potential role of pterostilbene in increasing lifespan both in male and female flies by mechanisms that seem to be different in the two sexes, highlighting the need to conduct nutraceutical supplementation studies on males and females separately in order to give more reliable results.

## 1. Introduction

Aging is a natural physiological process triggered by different molecular pathways and biochemical events that are promoted by both environmental and genetic factors. Aging is characterized by a time-dependent decline of functional capabilities and impaired stress resistance, that damage biomolecules compromising cellular homeostasis. The factors involved in aging are commonly referred to as the “hallmarks of aging”. Among them, oxidative stress and inflammation have been widely investigated. In 1956 Denham Harman [1] proposed“The Free Radical Theory of Aging” that has been updated in recent years to include the suggestion of a central role for reactive oxygen species (ROS) produced by mitochondria [2]. On the other hand, the term “inflammaging” has been created to indicate the significant contribution of low-grade, systemic inflammation to normal aging [3].

**Figure 1:**
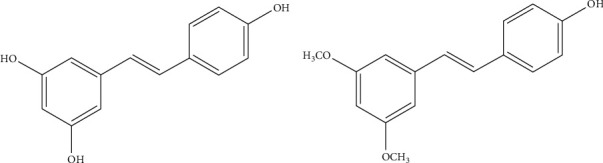
Chemical structure of A) resveratrol and B) pterostilbene.

The findings that the modification of environmental factors, such as diet, can increase lifespan, make natural dietary compounds extraordinary potential tools in the major healthcare challenge of delaying aging. Over the past 20 years, many studies have suggested that dietary polyphenols may exert beneficial effects as anti-aging compounds through the modulation of the hallmarks of aging, including inflammation, oxidative damage, cell senescence and telomere attrition [4–8]. Among the most prominent polyphenols, the stilbene resveratrol (5-[(E)-2-(4-hydroxyphenyl)ethenyl]benzene-1,3-diol) (Figure 1(a)) has gained widespread attention due to its ability to extend the lifespan of yeast, worms, and flies, and its ability to protect against age-related diseases such as cancer, Alzheimer's disease, and diabetes in mammals [9, 10].

Recently, pterostilbene (PTS) (Figure 1(b)) a dimethoxylated ether resveratrol derivative, has been gaining more and more attention thanks to its greater *in vivo* bioavailability and higher potential in modulating cognition and cellular stress with respect to resveratrol [11]. Both stilbenoids represent a group of natural phenolic compounds present in different types of plants and fruits such as grapes, tree wood, *Pterocarpus marsupium*, heartwood of sandalwood, leaves of *Vitis vinifera* and blueberries [12, 13].

Resveratrol has been more extensively characterized in terms of bioavailability and pharmacokinetics in human and animal studies compared to PTS. [9, 14–17]. What is known about PTS is that its structure, which differs from that of resveratrol by the presence of two methoxy (–OCH_3_) instead of two hydoxy (-OH) groups, may overcome the pharmacologic efficacy limitations observed in resveratrol (due to its poor absorption and rapid first-pass metabolism). When PTS is orally administered, it shows higher bioavailability (80% vs 20% of resveratrol), hepatic stability and total plasma levels when compare to resveratrol [18, 19]. Riche et al. [20] showed that PTS in a dose up to 250 mg per day is safe for humans and, thanks to its dimethoxy structure, that creates a favorable lipophilicity, has a higher membrane permeability and biological potency [21]. Furthermore, glucuronidation and sulfation processes are restricted by the methylation of the phenolic hydroxyl in PTS thus providing fewer conjugating sites compared to resveratrol and resulting in better metabolic stability.

In 2003, Howitz and colleagues [22] identified resveratrol as a potent activator of Sir2, the mammalian Sirt1 ortholog, capable of mimicking the effects of caloric restriction (CR) and regulating longevity in lower organisms such as worms and yeasts, while in *Drosophila melanogaster* there are contradictory results [23–25]. Besides NAD^+^ dependent histone deacetylase Sir2 [26], that controls enzymes regulating a large number of cellular pathways [27], there are other genes associated with longevity in *Drosophila*. Reduction in the activity of the nutrient-sensing insulin/IGF Signaling (IIS) pathway and the associated Target of Rapamycin (TOR) pathway results in one of the best understood interventions to extend flies lifespan [26]. The IIS pathway, in turn, intersects with a variety of other pathways that impact longevity such as the stress-responsive Jun-N-terminal kinase pathway, or the pathways that promote protein homeostasis and activate the mitochondrial unfolded protein response. Lifespan is also regulated by other mechanisms such as the loss of the mitochondrial co-transporter I'm Not Dead Yet (INDY) and the modulation of the mitochondrial electron transport chain [26].

To the best of our knowledge, there is no evidence that shows a direct effect of PTS on lifespan extension *in vivo*. Therefore, the present work sets out to study the potential role of PTS on longevity, oxidative stress and inflammation in *D. melanogaster* taking into consideration sex differences. We also carried out proteomic analysis on the whole *Drosophila* body, to have a deeper insight in the potential different effects of PTS supplementation on male and female flies.

## 2. Materials and Methods

### 2.1. Fly Strain, Rearing and Supplementation

The *Drosophila melanogaster* Canton S strain was a kind gift of Prof. Daniela Grifoni (University of l'Aquila, Italy). Flies were supplied with Formula 4–24 ® media (Carolina Biological, Burlington, NC, USA) and maintained at constant temperature (21°C) and humidity (60%) with a 12/12 h light–dark cycle. The diet contained: oat flour, soy flour, wheat flour, other starches, dibasic calcium phosphate, calcium carbonate, citric acid, niocinamide, riboflavin, sodium chloride, sodium iron pyrophosphate, sucrose, thiamine, mononitrate, brewer's yeast, emulsifier preservatives, mold inhibitor, food coloring [28]. Yeast pellets (*Saccharomyces cerevisiae*) were added to each tube after diet hydration. After eclosion, males and females were synchronized as reported in [29] and allowed to mate freely for two days before separating them according to sex [30]. PTS (Sigma-Aldrich s.r.l., Milan, Italy) was dissolved in DMSO (0.1%) and 50, 100, and 200 mM stocks were prepared and kept at -20°C until use. For supplementation, PTS was dissolved at different concentrations (50, 100 or 200 *μ*M) in the water used to soak 1.0 g of diet. A total of 20 flies were placed in each vial. The water used to prepare the control food contained 0.1% of DMSO.

### 2.2. Longevity Assay

Adult flies of both sexes were collected under FlyNap (Carolina Biological) anesthesia. A total of 800 male and 800 female fruit flies were randomly divided into 4 groups: CTR (control), PTS 50 (50 *μ*M PTS), PTS 100 (100 *μ*M PTS), and PTS 200 (200 *μ*M PTS). Every 2–3 days, the flies were transferred into vials containing fresh food and the number of living flies was counted. This was repeated until all flies died. Kaplan–Meier survival curves were generated for lifespan assessment.

### 2.3. Measurement of Body Weights

Body weights of male and female flies were recorded on days 15, 30, 45 and 60. Briefly, 20 flies in each group were anesthetized by FlyNap (Carolina Biological) and then weighed on a balance. The mean body weights of the flies in each group were calculated.

### 2.4. CApillary FEeder (CAFE) Assay

Food intake was measured using the capillary feeder method (CAFE) as reported in [31] with minor modifications. Flies were held in shortened culturing vials of 7 cm in length. Cotton balls, soaked with 500 *μ*L of water, were inserted at the bottom of tubes for humidity, the tubes were then parafilmed to reduce evaporation and keep a high level of humidity inside the vials. Microcapillary tubes were inserted into the tubes through a 200 *μ*L pipette tip in the foam plug. Four microcapillary tubes were used per vial and filled with 100 *μ*M PTS (PTS) or 0.1% DMSO diluted in 2.5% sucrose (CTRL). The assay was performed on flies never supplemented (3 days old male and female flies) or on flies supplemented with PTS or 0.1% DMSO for 15 days. At each time point 2 groups for sex were considered: PTS and CTRL. On the day of the experiment, flies were weighted, starved for two hours, and then separated into CAFE vials, with eight flies for vial (40 flies/sex/condition; n =80 flies). To account for evaporation of the liquid food, three vials were set up with feeding capillaries but without flies. Fly consumption was evaluated after four hours measuring the amount of liquid consumed from the microcapillary tube (in mm) as described by Fiocca et al. [32] and data were reported as *μ*L/mg of fly.

### 2.5. Paraquat Toxicity

Oxidative stress resistance was measured using paraquat (1,1′-dimethyl-4,4′-bi-pyridinium dichloride, Sigma-Aldrich s.r.l.) as stress inducer. Briefly, 15 days-old flies were subjected to starvation for 2 hours then transferred to parafilm-sealed vials (ten flies per vial) containing capillaries filled with 2.5 mM paraquat prepared with 2.5% sucrose solution. For each condition, 50 flies per sex were tested. Mortality was scored every 6 hours until all flies died and expressed as percentage of survival.

### 2.6. Ethanol Toxicity

Ethanol toxicity was evaluated as reported by Niveditha [33] with some modifications. Briefly, 15 days-old flies (supplemented and CTR) were first subjected to starvation for 2 h then transferred to parafilm-sealed vials (ten flies per vial) containing Whatman filter paper discs (diameter 2 cm) soaked with 2.5% sucrose solution and 17.5%, ethanol (Carlo Erba, Milano, Italy). Mortality was scored every 2 hours until all flies died and expressed as percentage of survival. For each condition, 50 flies per sex were tested.

### 2.7. DCFH-DA Assay

ROS content was evaluated using 2′-7′-dichlorodihydrofluorescein diacetate (DCFH-DA). Groups of 25 flies were mechanically homogenized in 1 mL of 0.1 M PBS. The homogenate was centrifuged at 6,000 rpm for 10 min and 200 *μ*L of supernatant was incubated with 50 *μ*M DCFH-DA (Sigma-Aldrich s.r.l.) at 37°C. The fluorescence was measured each 10 minutes in a FLUOstar Omega plate reader (BMG Labtech, Ortenberg, Germany) using ex/em wavelength of 485/520 nm. ROS levels were quantified as increase in DCF fluorescence through the time normalized by protein and expressed as increase/min/*μ*g protein. The protein concentration in the homogenate was quantified by Bradford method using bovine serum albumin (Sigma-Aldrich s.r.l) as standard. Each condition was the results of six replicates and was expressed as mean ± SEM.

### 2.8. Total Antioxidant Capacity

To assess the total antioxidant capacity of flies, the 2, 2′-azino-bis (3-ethylbenzothiazoline-6-sulphonic acid, Sigma-Aldrich s.r.l.) (ABTS) assay, as reported by Re et al. [34] and modified for application to a 96-well microplate, was used.

Flies were mechanically homogenized in 1 mL of 0.1 M PBS on ice. The homogenate was centrifuged at 6,000 rpm for 10 min and the supernatant was used for the assay.

The ABTS^•+^ solution was freshly prepared by the oxidation of ABTS (10 mg) by MnO₂ (0.75 g) in the presence of water (4 mL), followed by 30 min at room temperature. The working solution was obtained by diluting the previous mixture with H_2_O to have an absorbance around 1 at 734 nm.

For the assay, 200 *μ*L of the working solution was added to 50 *μ*L aliquot of progressively diluted samples and Trolox as a standard. The absorbance of each well was measured after 15 min incubation at 734 nm.

The protein concentration in the homogenates was quantified by Bradford method using bovine serum albumin as standard. Each condition was the results of six replicates and was expressed as mean ± SEM. The total antioxidant capacity of flies was compared to Trolox used as positive control and expressed as mg of Trolox-equivalent antioxidant capacity for mg of homogenate protein (TEAC).

### 2.9. RNA Extraction

RNA was extracted from the whole bodies of flies using RNeasy Mini Kit (QIAGEN GmbH, Hilden, Germany). NanoVue Spectrophotometer (GE Healthcare, Milano, Italy) was used to measure the yield and purity of the RNA. Only samples with ratios A260/A280>1.8 were used.

### 2.10. Analysis of mRNA Levels by Reverse Transcriptase Polymerase Chain Reaction

For each sample, 1 *μ*g of total RNA was reverse transcribed to obtain cDNA using iScript cDNA Synthesis Kit (Bio-Rad Laboratories, Hercules, CA, USA) following the manufacturer's instructions. The subsequent polymerase chain reaction (PCR) was performed in a total volume of 10 *μ*L containing 2 *μ*L of dH_2_O RNAsi free, 2.5 *μ*L (12.5 ng) of cDNA, 5 *μ*L SsoAdvanced Universal SYBR Green Supermix (Bio-Rad Laboratories), and 0.5 *μ*L (500 nM) of each primer. The primers used were purchased from Sigma-Aldrich s.r.l. and they are reported in Table 1; RPL32 was used as reference gene.

### 2.11. Proteomic Analysis

For proteomic analysis, proteins were extracted from total body of male and female flies supplemented with 100 *μ*M PTS for 15 days using 8 M urea, 2 M thiourea, 4% CHAPS and 60 mM dithiothreitol (DTT) extraction solution. Briefly, bodies of flies were resuspended in extraction solution using a microtube pestle and sonicated 1 min for 5 times in an ultrasonic bath. After incubation for 1 h at room temperature, samples were centrifuged at 16,000 x g for 10 min to remove undissolved material. Protein concentration was determined using the Pierce Protein Assay (Thermo Fisher Scientific, Waltham, MA, USA) and bovine serum albumin was used as standard. Two-dimensional gel electrophoresis (2DE) was carried out as previously described [35]. Briefly, 200 *μ*g of proteins were filled up to 350 *μ*L in rehydration solution. Isoelectrofocusing (IEF) was performed using 18 cm Immobiline Dry Strips (GE Health Care Europe, Uppsala, Sweden) with a nonlinear pH 3-10 gradient. IEF was carried on the Ettan IPGphor Cup Loading Manifold (GE Healthcare). After IEF, the strips were equilibrated during two steps of 15 min each in equilibration buffer (0.05 M Tris, 6 M urea, 2% SDS, 20% glycerol) with 1% DTT in the first incubation or 2.5% iodoacetamide in the second one. The subsequent electrophoresis (Sodium Dodecyl Sulphate-Polyacrylamide Gel Electrophoresis; SDS-PAGE) was carried out by transferring the proteins to 12% polyacrylamide, running at 16 mA per gel and 10°C for about 16 h, using the Protean® Plus Dodeca Cell (Bio-Rad). The gels were stained with Ruthenium II tris (bathophenanthroline disulfonate) tetrasodium salt (Cyanagen, Bologna, Italy) (RuBP). ImageQuant LAS4010 (GE Health Care) was used for the acquisition of images. The analysis of images was performed using Same Spot (v4.1, TotalLab, Newcastle Upon Tyne, UK) software.

Gel spots were excised and in-gel digested [36]. Peptides were lyophilized and resuspended in 2% acetonitrile (ACN), 0.1% formic acid (FA) and 97.9% water.

### 2.12. Mass Spectrometry Analysis

NanoLiquid Chromatography coupled tandem mass spectrometry analysis (nLC-ESI-MS/MS) was achieved on an analytical platform comprising an UltiMate3000 RSLCnano System interfaced with an Orbitrap Fusion Tribrid mass spectrometer through a nanoESI source (EASY-Spray NG) (Thermo Fisher Scientific, Milan, Italy) operating in positive ion mode, as already described [37] with minor modifications. After trapping and desalting on a micro-precolumn, a 15 min linear gradient starting from 95% solution A (0.1% FA in water) to 25% solution B (0.1% FA in ACN) allowed peptides' separation on an EASY-Spray PepMap RSLC C18 column (2 *μ*m particle size, 100 Å pore size, 75 *μ*m i.d. x 25 cm length, Thermo Fisher Scientific) for a total run of 40 min.

Precursor ions were recorded by the Orbitrap detector and fragments (MS/MS) ions by the Ion Trap at rapid scan rate. Twenty most abundant multiple-charged (2^+^ – 7^+^) precursor ions, detected within the range of 275−1,750 m*/z*, were subjected to fragmentation by Collision-Induced Dissociation (CID) with dynamic exclusion of 45 s and using 35% normalized collision energy. The signal intensity threshold for MS/MS was set to 5 × 10^3^. Proteome Discoverer software (version 2.4, Thermo Fisher Scientific) was used to process the raw data searching the fruit fly UniProtKB reference proteome (*Drosophila melanogaster* database, ID: UP000000803, release: 2021_01, 22,117 proteins) to which 39 common contaminant sequence entries were appended. Protein identification search parameters were set as follows: chosen enzyme: trypsin with a maximum of 1 missed cleavage per peptide; variable modification: oxidation of methionine and acetylation of lysines at protein N-terminus; static modification: carbamidomethylation of cysteine; mass tolerance of precursor: 10 ppm; fragment mass tolerance: 0.6 Da. A threshold of two peptides with high confidence peptide-spectrum matches was applied for protein identification.

### 2.13. Statistical Analysis

Each experiment was performed at least three times, and all values are represented as means ± SEM. Student's t-test or one-way ANOVA were used to compare differences among groups followed by Bonferroni's test (Prism 5, GraphPad Software, San Diego, CA). Values of p <0.05 were considered statistically significant. Survival curves were prepared by Kaplan-Meier survival analysis and analyzed using the OASIS2 software [38]. For proteomic experiments, statistical analysis was based on the normalized volume of each spot calculated by the software. Comparison analysis was performed between PTS-treated and control images in both females and males, using One-way ANOVA. Spots that exhibited ratio ≥1.2 or ≤0.83, p-value <0.05 and q value <0.05 were taken into consideration for further protein identification. Bioinformatic analysis was carried out using ShinyGO v.0.66 software to obtain interactive plots showing the relationship between enriched pathways whereas heat map was build using NG-CHM GUI 2.20.2 software [39].

## 3. Results

### 3.1. Effect of PTS on Longevity

**Figure 2:**
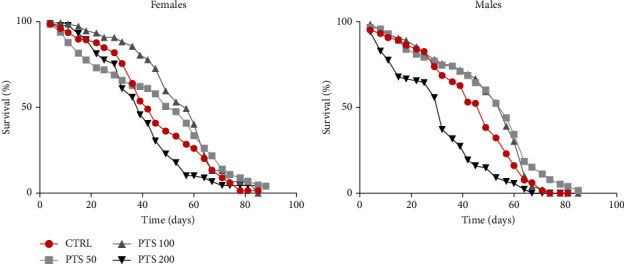
Survival assay of adult female and male flies. Flies were supplemented with 50, 100, and 200 *μ*M PTS lifelong. Data are presented as percentage of survival of flies as function of time (in days). The Kaplan–Meier test was used to detect the significant differences among the fourth groups of flies.

To examine the pro-longevity effect of PTS, male and female Canton S flies were reared on standard diet supplemented with different concentrations of PTS (50, 100, and 200 *μ*M) lifelong (Figure 2). DMSO vehicle (0.1%) has been added to control groups. DMSO did not influence fly longevity as no significant differences have been observed between control groups reared in the presence or absence of 0.1% DMSO both in male and female flies (data not shown). Moreover, this concentration of DMSO has been used by different authors as control vehicle [40–42]. A significant increase in mean lifespan was observed in 50 *μ*M PTS supplemented male flies compared to control flies (14% increase, p <0.0034), while mean lifespan of female flies treated with 50 *μ*M PTS was comparable to that of control flies (Figure 2). 100 *μ*M PTS was effective both in male and female flies leading to a significant increase of mean lifespan of 12% (p <0.0196) and 20% (p <0.0072), respectively. Of note, 200 *μ*M PTS had a negative effect in both sexes as it significantly reduced mean lifespan in respect to control flies.

**Figure 3:**
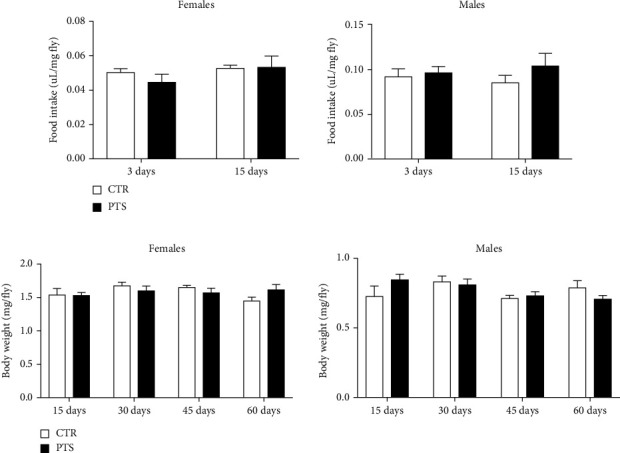
Food intake and body weights of *D. melanogaster* supplemented with PTS. (a). Flies were not supplemented with PTS or supplemented with 100 *μ*M PTS for 15 days before CAFE assay. (b). Flies were supplemented with 100 *μ*M PTS for 15, 30, 45, and 60 days before body weight measurement. Each bar represents the mean ± SEM. Data were analyzed by Student's t-test comparing each supplementation to the corresponding control.

To verify that the improvement of the mean lifespan was due to PTS supplementation itself and not to CR induced by PTS off-flavor, food intake was evaluated by CAFE assay in flies before supplementation and after 15 days of PTS supplementation (Figure 3(a)). Interestingly, PTS did not influence food intake in female and male flies both before and after PTS supplementation. Of note, male flies had a higher food intake in respect to female flies. In particular, PTS supplemented male flies had a significant higher food intake in respect to female PTS supplemented flies both at 3 days and 15 days (p <0.0014 and p <0.0341, respectively). Body weights of flies supplemented with 100 *μ*M PTS were recorded on days 15, 30, 45 and 60 (Figure 3(b)) to further confirm that PTS did not modify food intake throughout fly life. No differences in body weights were observed both in female and male flies at any time points, suggesting an equal food intake in control and PTS supplemented groups.

**Figure 4:**
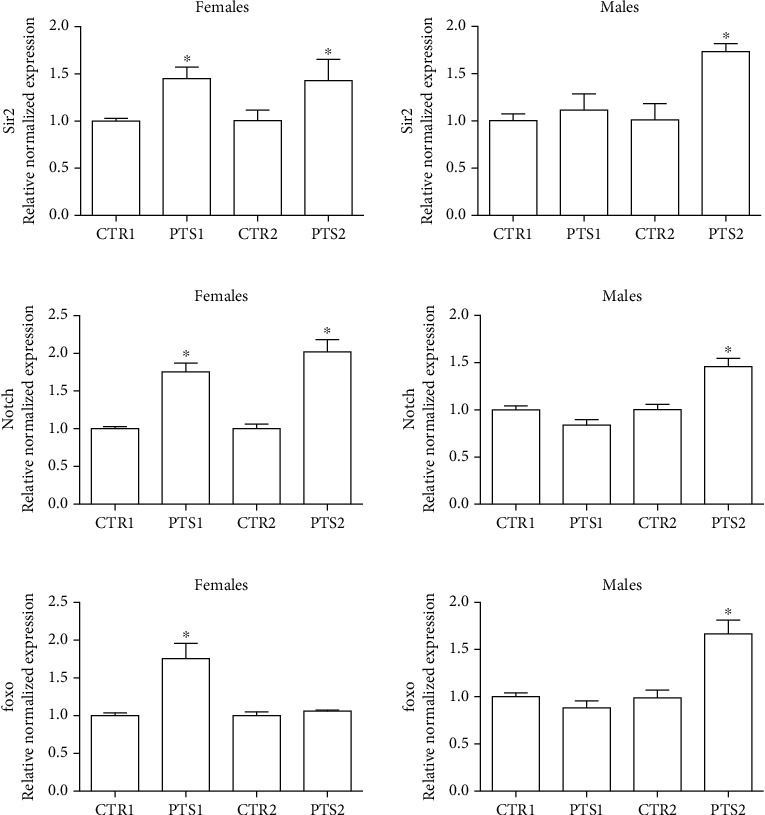
Expression of genes related to longevity in D. melanogaster supplemented with PTS. Flies were supplemented with 100 *μ*M PTS for 15 days (PTS1) or 60 days (PTS2). Total RNA was isolated, and the mRNA levels of A) Sir2, B) Notch, and C) foxo were quantified using RT-PCR normalized to RPL32 reference gene as reported in Materials and Methods. Triplicate reactions were performed for each experiment. Each bar represents the mean ± SEM of three independent experiments. Data relative to 15 days and 60 days were grouped into one graph but analyzed separately by Student's t-test. ∗p <0.05 with respect to the corresponding controls, CTR1 or CTR2.

To better clarify, at a molecular level, the effect of PTS on the lifespan of male and female flies, the expression of 3 genes related to longevity were investigated. As 100 *μ*M PTS supplementation was effective both in males and females, and in males showed comparable effects to 50 *μ*M PTS, 100 *μ*M PTS supplementation was used in the following experiments. Flies were supplemented with PTS for 15 days (PTS1) or 60 days (PTS2) and the expression of Sir2, foxo, and Notch was evaluated by RT-PCR (Figure 4).

Sir2, a member of the Sirtuin family of protein acylases, deacetylates lysine residues within many proteins and has been implicated in the extension of longevity in *D. melanogaster* [43]. In Figure 4(a) the expression levels of Sir2 are reported. Sir2 was significantly upregulated in female flies both after 15 days and 60 days, meanwhile in male flies the gene was overexpressed only after 2-month supplementation. As it has been recently demonstrated that Sir2 is a positive regulator of the Notch pathway in *Drosophila* [44], we also investigate the effect of a short and long term supplementation of PTS on the expression of Notch in male and female flies (Figure 4(b)). Interestingly, the obtained data were perfectly in agreement with the results on Sir2 expression. In fact, in female flies, both the short- and long-term supplementations with PTS were able to up-regulate Notch expression. In male flies only the 2-month supplementation led to a significant Notch over-expression. In *D. melanogaster*, FOXO, the orthologous of the nematode DAF-16/FoxO and mammalian FOXO3A, has been shown to be a key transcriptional regulator of the insulin pathway that modulates growth and proliferation, and the increase of its activity in certain tissues is sufficient to extend fly lifespan [45]. In our study, PTS triggered a significant foxo up-regulation after 15 days supplementation in female flies and after 2-month supplementation in male flies (Figure 4(c)).

### 3.2. Antioxidant Effect of PTS

**Figure 5:**
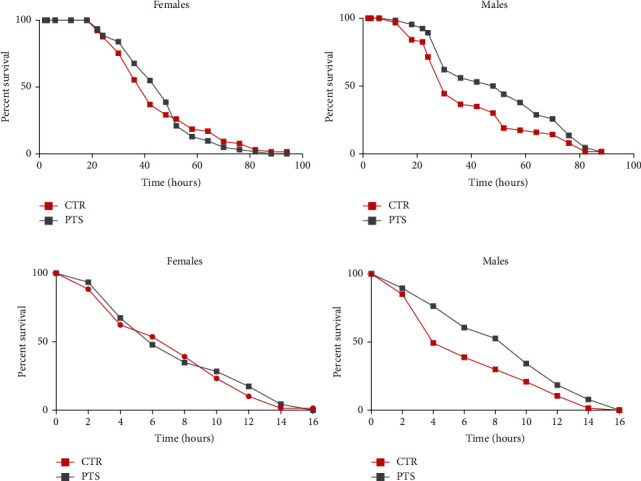
Survival assay of adult female and male flies after oxidative stress induction. Flies were supplemented with 100 *μ*M PTS for 15 days, then were exposed to 2.5 mM Paraquat **(A, B)** or 17.5% ethanol **(C, D)**. Data are presented as percentage survival of flies as function of time (in hours). The Kaplan–Meier test was used to detect the significant differences among the two groups of flies.

**Figure 6:**
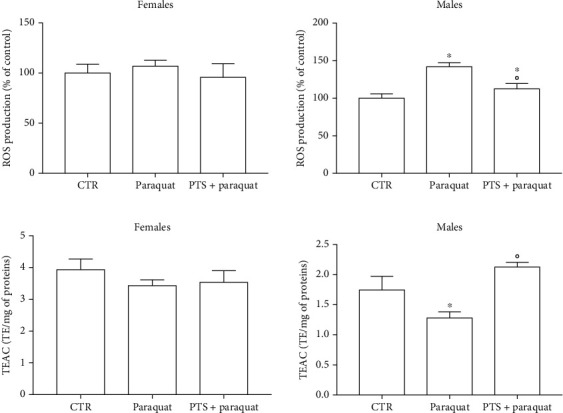
Redox state of flies supplemented with PTS and exposed paraquat. Flies were supplemented with 100 *μ*M PTS for 15 days, then were exposed to 2.5 mM paraquat. A. ROS production was measured with the peroxide-sensitive probe DCFH-DA B. Total radical scavenging capacity was evaluated by ABTS assay. Each value represents mean ± SD. Data were analyzed by One-way ANOVA followed by Bonferroni's test. ∗p <0.05 with respect to CTR; °p <0.05 with respect to Paraquat.

**Figure 7:**
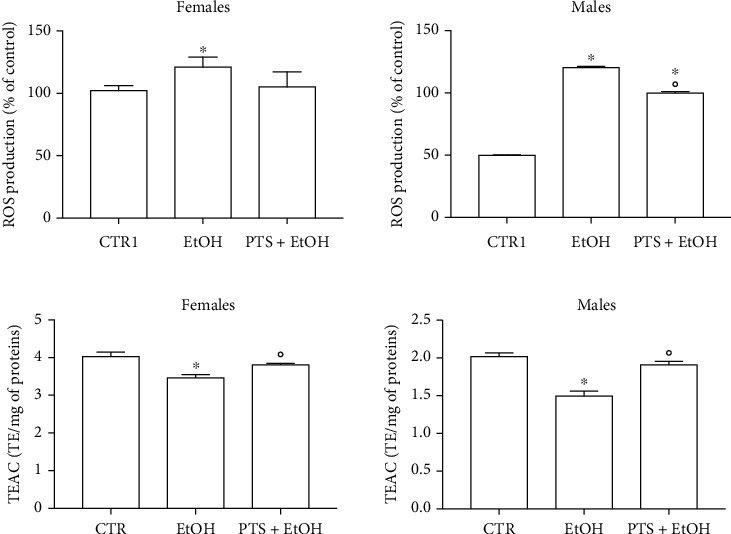
Redox state of flies supplemented with PTS and exposed to ethanol. Flies were supplemented with 100 *μ*M PTS for 15 days, then were exposed to 17.5% ethanol (EtOH) for 4 h. A. ROS production was measured with the peroxide-sensitive probe DCFH-DA B. Total radical scavenging capacity was evaluated by ABTS assay. Each value represents mean ± SD. Data were analyzed by One-way ANOVA followed by Bonferroni's test. ∗p <0.05 with respect to CTR; °p <0.05 with respect to EtOH.

To study the antioxidant effect of PTS, flies were supplemented with 100 *μ*M PTS for 15 days before the induction of oxidative stress. As we mentioned above, we chose this PTS concentration because, it was the most effective in increasing lifespan in both sexes. Oxidative stress was induced by 2.5 mM paraquat or 17.5% ethanol exposure in both male and female flies [33, 46–49]. PTS did not show any protective activity against both paraquat and ethanol-induced damage in female flies since the survival curves of control and PTS supplemented flies were comparable (Figure 5(a)–5(c)). On the contrary, 100 *μ*M PTS significantly increased the percentage of survival in male flies exposed to both paraquat and ethanol (p <0.017 and p <0.0284, respectively). The redox state of flies supplemented with PTS and exposed to paraquat or ethanol was investigated evaluating both the production of ROS and the total radical scavenging capacity (Figures 6 and 7). In male flies paraquat significantly increased ROS production with respect to control, and PTS administration was able to partially counteract this effect significantly reducing ROS production compared to paraquat but maintaining ROS production to levels significantly higher in respect to controls (Figure 6(a)). These data are strengthened by the results on the antioxidant capacity of male flies, as paraquat significantly reduced the total radical scavenging capacity in respect to controls, meanwhile PTS reverted this effect significantly increasing the endogenous antioxidant levels to value comparable to controls (Figure 6(b)). In female flies paraquat did not modify endogenous ROS production (Figure 6(a)). Slight reduction of the total radical scavenging capacity is observable both in females exposed to paraquat and in those pre-treated with PTS even if these results are not statistically significant (Figure 6(b)).

Ethanol significantly and strongly increased ROS production (Figure 7(b)) and decreased the total radical scavenging capacity (Figure 7(c)) in male flies. PTS supplementation significantly decreased ROS levels and increased the total radical scavenging capacity in respect to male flies exposed to ethanol, but only the total radical scavenging capacity reached levels comparable to control flies. In agreement with the data obtained with paraquat, in female flies, ethanol induced a lower increase of ROS levels in respect to male flies. PTS triggered a slight reduction of ROS levels with intermediate values between control and ethanol stressed flies. Total radical scavenging capacity was significantly reduced by ethanol exposure in female flies and PTS was able to significantly increase this value in respect to ethanol treated flies.

**Figure 8:**
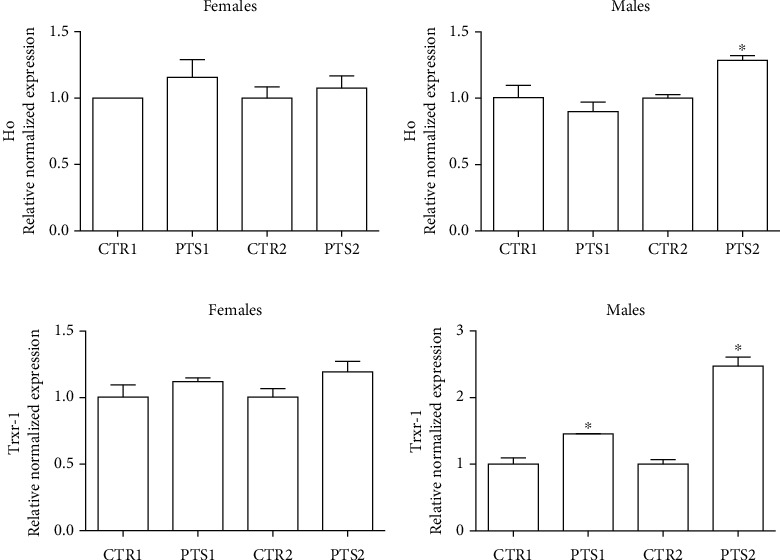
Expression of antioxidants enzymes in *D. melanogaster* supplemented with PTS. Flies were supplemented with 100 *μ*M PTS for 15 days (PTS1) or 60 days (PTS2). Total RNA was isolated, and the mRNA level of A) Ho and B) Trxr-1 was quantified using RT-PCR normalized to RPL32 reference gene as reported in Materials and Methods. Triplicate reactions were performed for each experiment. Each bar represents the mean ± SEM of three independent experiments. Data relative to 15 days and 60 days were grouped into one graph but analyzed separately by Student's t-test. ∗p <0.05 with respect to controls, CTR1 and CTR2.

To further investigate the antioxidant activity of PTS, the expression of 2 antioxidant genes, heme oxygenase (Ho) and thioredoxin reductase 1 (Trxr-1), was evaluated. Flies were supplemented with PTS for 15 days (PTS1) or 60 days (PTS2) and the expression of Ho and Trxr-1 was measured by RT-PCR (Figure 8).

In agreement with the previous data on oxidative stress, PTS did not influence the expression of both the enzymes in female flies. On the other hand, in male flies, PTS supplementation significantly up-regulated Trxr-1 at both supplementation times, and triggered Ho over-expression at the second time point.

### 3.3. Anti-Inflammatory Effect of PTS

**Figure 9:**
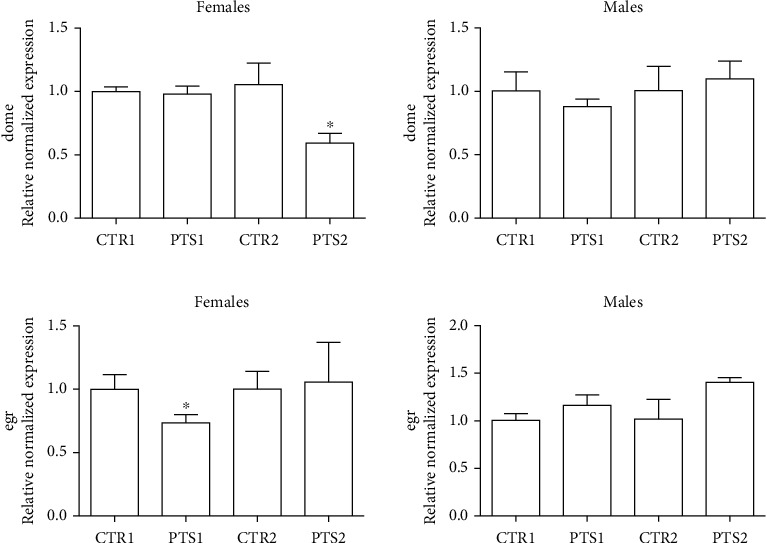
Expression of inflammatory cytokines in *D. melanogaster* supplemented with PTS. Flies were supplemented with100 *μ*M PTS for 15 days (PTS1) or 60 days (PTS2). Total RNA was isolated, and the mRNA level of A) dome and B) egr was quantified using RT-PCR normalized to RPL32 reference gene as reported in Materials and Methods. Triplicate reactions were performed for each experiment. Each bar represents the mean ± SEM of three independent experiments. Data relative to 15 days and 60 days were grouped into one graph but analyzed separately by Student's t-test. ∗p <0.05 with respect to controls, CTR1 and CTR2.

One of the most recent theories on aging focuses on the activation of a subclinical, chronic low-grade inflammation that occurs within this process, named “inflammaging” [3]. Even if a lot of studies have demonstrated PTS anti-inflammatory activity in different model systems, no study investigated the anti-inflammatory effect of PTS in *D. melanogaster*. On this basis, female and male flies were supplemented with 100 *μ*M PTS for 15 days (PTS1) or 60 days (PTS2) and the expression of two pro-inflammatory genes, domeless (dome) and eiger (egr), were measured by RT-PCR (Figure 9). In particular, dome is a signal-transducing receptor with most similarities to the IL-6 receptor family [50], whereas egr is the fly ortholog of TNF*α* [51]. In female flies, PTS significantly reduced the expression of dome at 60 days supplementation and the expression of egr at 15 days supplementation. No effect has been observed in male flies.

### 3.4. Proteome Changes Induced by PTS Supplementation

**Figure 10:**
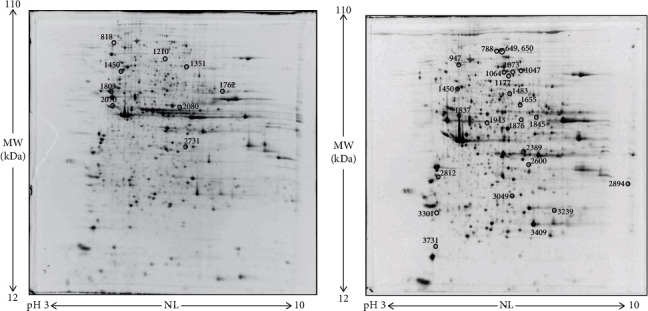
Representative 2DE images of protein extracts of bodies of both female (a) and male (b) flies supplemented with 100 *μ*M PTS for 15 days. Protein extracts were separated in a 3–10 nonlinear gradient. SDS-PAGE was performed using 12% acrylamide. Gels were stained with ruthenium. Spot numbers indicate all the proteins identified by nLC-ESI-MS/MS and refer to the number reported in Table 2.

**Figure 11:**
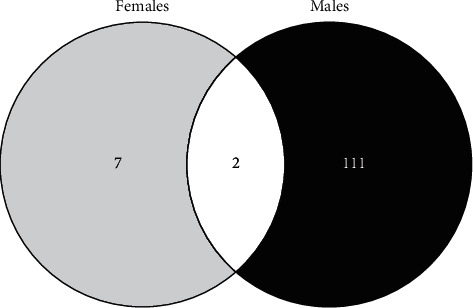
Venn diagram highlighting the distribution of differentially expressed spots in protein extracts obtained by PTS supplemented flies compared to control flies. Both unique and overlapping spots are reported as actual number (Venny 2.0.2).

**Figure 12:**
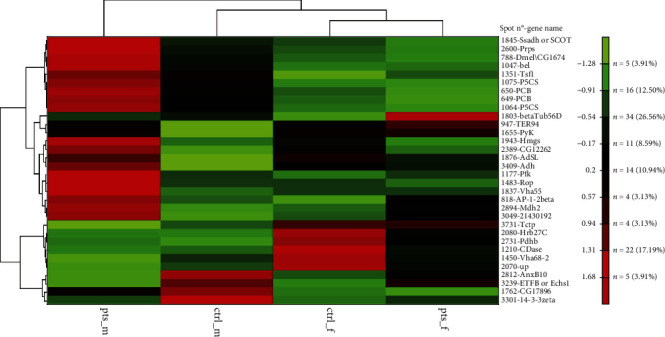
Clustered heat map of differentially expressed proteins after PTS supplementation in female and male samples. Heat map was generated using NG-CHM GUI 2.20.2 software. Z-norm transform was used to normalize the row values (mean of normalized optical densities of spots, n =3) and resulting transform data matrix used to build the heat map. A hierarchical ordering method was applied to clustered rows and columns. Euclidean distance metric was applied to hierarchically clustered rows and columns. Data matrix distribution values range from -1.72 to +1.72. The brighter the color, the more intense the changes are. ctrl_m: male control; ctrl_f: female control; pts_m: males treated with PTS; pts_f: females treated with PTS.

**Figure 13:**
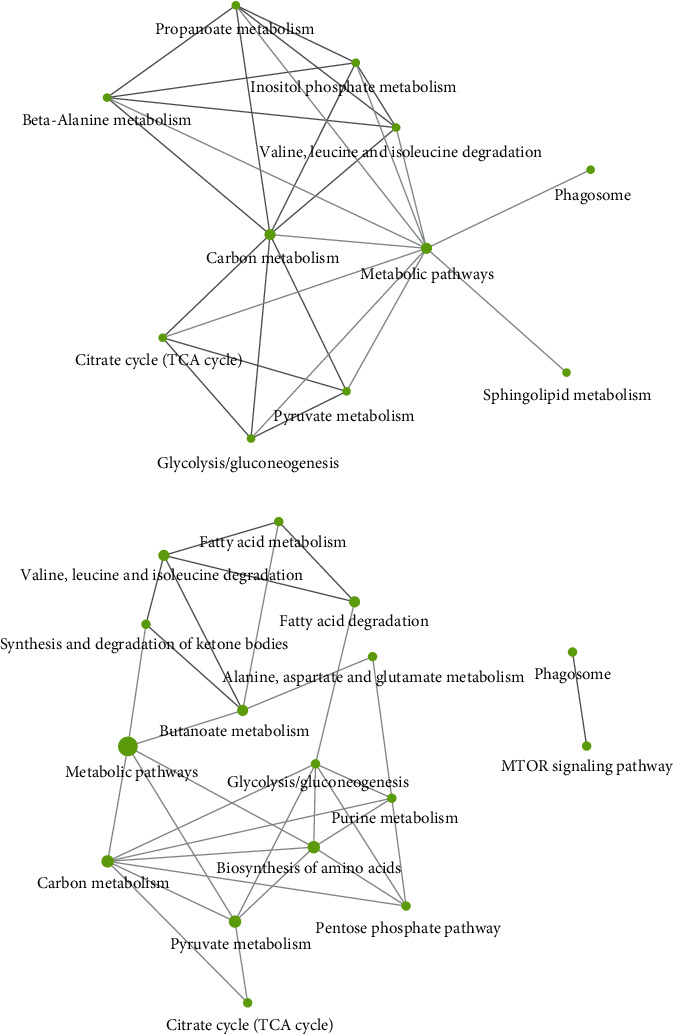
KEGG Pathway analysis of differentially expressed proteins both in females (a) and males (b) *D. melanogaster*. ShinyGO v.0.66 software was used to obtain interactive plots which show the relationship between enriched pathways. Two pathways (nodes) are connected if they share 20% (default) or more genes. Darker nodes are more significantly enriched gene sets. Bigger nodes represent larger gene sets. Thicker edges represent more overlapped genes.

To understand why PTS supplementation for 15 days counteracted oxidative stress in male but not in female flies, we decided to look at the proteome changes that may be aroused from this supplementation in the two sexes.  Figure 10 shows representative 2DE images of protein extracts of bodies of both female (A) and male (B) flies supplemented with 100 *μ*M PTS for 15 days. Comparative analysis was performed to investigate the effects of PTS in respect to control both in female and male groups. One-hundred and thirteen and nine spots were found differentially expressed (ratio ≥1.2 or ≤0.83, p <0.05, q value <0.05) in male and female flies supplemented with PTS in respect to controls, respectively. For males, only 23 proteins were identified, of which 19 were significantly induced in respect to control flies and 4 were significantly lower than control flies. Venn diagram (Figure 11) illustrates the number of spots differentially expressed which are common and exclusive between the two sexes. Of the nine proteins modulated by PTS in female flies, only two were in common with the proteins differentially expressed in males. Spots of interest were subsequently subjected to nLC-ESI-MS/MS analysis and identified.  Table 2 shows the list of identified proteins together with their MW, pI, peptides and coverage values of MS/MS, ratio and p values. Moreover, normalized mean values of optical density of the identified differentially expressed spots were analyzed using Next-Generation Clustered Heat Map to generate a clustered heat map (Figure 12). In the heat map it is possible to appreciate the higher number of up-regulated proteins after PTS treatment in male flies compared to females. In particular, as suggested by gene ontology analysis, the major changes observed in the males involve proteins belonging to metabolic processes (i.e. small molecule metabolic process, carboxylic acid, oxoacid, ATP, and phosphorus metabolic processes) (Figure 13(b)). Very few changes were observed in the females and essentially related to proteins involved in homeostasis processes and to a lesser degree to metabolic processes (Figure 13(a)).

## 4. Discussion

Recent studies suggest that PTS can be considered a potential candidate as an anti-aging agent thanks to its ability to modulate different hallmarks of aging, including oxidative damage, inflammation, telomere attrition, and cell senescence [52]. The potential health benefits of PTS on lifespan extension are supported by different studies that investigated the effect of blueberry on model organisms. In *Caenorhabditis elegans*, blueberry polyphenols increased lifespan and thermo-tolerance [6] and blueberry extracts contributed to lifespan prolongation of *D. melanogaster* by the up-regulation of the antioxidant enzymes SOD and catalase [53]. These studies hypothesize that PTS plays a role in increasing longevity but, to the best of our knowledge, no author has so far demonstrated a direct effect of PTS on this parameter. Here, we investigated the effect of PTS supplementation on *Drosophila* longevity and its potential mechanisms of action.

Our data show that PTS supplementation increases the mean lifespan of male and female flies at specific concentrations. In particular, 100 *μ*M PTS was effective in both male and female flies, 50 *μ*M increased mean lifespan only in male flies whereas 200 *μ*M PTS evidenced a toxic effect on both sexes. Interestingly, PTS supplementation did not modify the amount of food intake in *Drosophila*, so the observed effect is not due to a potential CR induced by PTS off-flavor. Rather, these results could be related to a hormetic effect, a biphasic dose-response mechanism characterized by a low-dose beneficial effect and a high-dose toxic effect *[*54*]*. In agreement with our results, different natural products with a pro-longevity effect have been shown to reduce the lifespan of animals when supplemented at high doses *[*55*]*. PTS is structurally like resveratrol as both are monomeric stilbenes with the only difference that PTS has two methoxy (–OCH_3_) groups and one hydroxyl (−OH) group, while resveratrol has three −OH groups (Figure 1). Different studies investigated the effect of resveratrol supplementation on D. melanogaster lifespan with apparently contradictory results. Staats et al. [56] did not observe any effect on lifespan after a 500 *μ*M resveratrol supplementation in D. melanogaster, meanwhile Abolaji et al. [57] showed that resveratrol extended lifespan in a dose dependent manner up to a concentration of 60 mg/kg diet. Interestingly, 120 mg/kg diet did not modify lifespan when compared to control flies as observed by Staats et al., suggesting that only specific resveratrol concentrations are effective in prolonging lifespan. These data agree with our observations on the effect of PTS on the mean lifespan of *D. melanogaster* where only one dose in females (100 *μ*M) and two doses in males (50 and 100 *μ*M) where able to increase this parameter. Moreover, we observed a higher food intake in male flies compared to female flies and this could explain why 50 *μ*M PTS supplementation increased mean lifespan of male flies but was not effective on female flies.

As resveratrol has been demonstrated to be effective in increasing lifespan of different model organisms through the up-regulation of Sirt1 [52], we hypothesized that PTS could act on the same molecular target in *Drosophila*. It has been shown that Sirt1, a (NAD+)-dependent deacetylase, has a role in lifespan extension, stress resistance and apoptosis reduction [58, 59]. Sirt1 regulates a great number of downstream molecules, including p53, Foxo1, Foxo3, Foxo4, E2F1, and Notch [44, 60]. Our data demonstrate that PTS supplementation up-regulates the *Drosophila* Sirt1 homolog, Sir2, already after 15 days in females, and in both sexes after 60 days. Of note, to our knowledge, no other study demonstrated that PTS increases Sirt1 in healthy individuals undergoing a physiological aging process. In fact, the research demonstrating PTS ability to increase Sirt1 expression have been carried out in different pathological conditions in which Sirt1 resulted down-regulated in respect to healthy individuals.

To further investigate the effect of PTS on the mean lifespan in *D. melanogaster* we measured the expression of two downstream target of Sir2, Notch and foxo. The Notch signaling pathway is highly conserved among species from *Drosophila* to humans. It has a fundamental role in adult central nervous system for neural plasticity and triggers neural differentiation during development [61]. Notch is a membrane-bound transcription factor that is released to the nucleus by a two-step cleavage mechanism [62]. The second cleavage is carried out by Presenilin and knockout of Presenilin causes the impairment of synaptic plasticity and memory formation in mice [63]. Moreover, it has been observed an impairment in spatial learning and memory of mice carrying a heterozygous mutation of Notch [64]. Presente et al. [61] observed that *D. melanogaster* with a selective loss of Notch function in adulthood show a syndrome that includes loss of flight and premature death from unknown causes. In our study, PTS supplementation triggered a significant up-regulation of Notch with the same regulatory pattern of Sir2, suggesting, as previously underlined, a strong crosstalk between these two proteins.

Foxo is a transcription factor acting as downstream effector of insulin signaling and its activity has been observed to be strictly related with stress resistance and lifespan extension in many organisms including humans [65]. In our model system, PTS led to an increase of foxo expression in female flies only after 15 days supplementation and in male flies only after 2-month supplementation, suggesting a potential role in modulating longevity of both sexes, but indicating that PTS can have different effects in male and female flies.

There are many factors that cause aging, among which oxidative stress and inflammation have been demonstrated to play a fundamental role [66]. Oxidative stress is an imbalance between the production and the removal of reactive oxygen species. According to the oxidative stress theory of aging, the functional impairment typical of elderly is connected to the accumulation of structural alterations due to the oxidative damage to macromolecules by ROS [67]. On these bases, we verified if PTS boosts the *Drosophila* antioxidant system making flies more resistant to oxidative stress. To mimic this condition, we exposed flies to paraquat [48, 49] and ethanol [47, 68]. Interestingly, after exposure to acute ethanol doses, fruit flies show behaviors similar to those observed in humans and mammalian models [69].

A PTS supplementation for 15 days was able to counteract oxidative stress only in male flies, while it was not effective in female flies, evidencing, once again, the different effect of PTS on the two sexes. Interestingly, our data obtained by proteomic analysis indicate that PTS is able to significantly increase alcohol dehydrogenase (ADH) expression only in male flies, supporting the positive PTS activity against ethanol in male flies but not in female flies. In D. melanogaster, ADH metabolizes more than 90% of ethanol and in ADH deficient flies a higher toxicity of ethanol has been observed [70]. Of note, in the first 24 h, paraquat induced a higher mortality in male flies in respect to female flies (p <0.0063). Our results are in agreement with the data of Krůček et al. [71] that observed a higher mortality induced by 25 mM Paraquat in male flies in respect to female flies. This could be ascribed to a different expression of the endogenous antioxidant enzymes in the two sexes. In fact, as demonstrated by Niveditha et al. [33], in female flies the activity of the antioxidant enzymes SOD and catalase is significantly higher in respect to male flies. These data are in agreement with our results that evidenced a higher antioxidant capacity of females flies (~3.9 mg TE/mg protein) in respect to male flies (~1.7 mg TE/mg protein).

To have a better insight on the antioxidant mechanisms of PTS in *Drosophila* we investigated the effect of PTS on the expression of two antioxidants enzymes, Ho and Trxr-1. We decided to investigate these two antioxidant enzymes because Ho has been demonstrated to be upregulated by PTS in both cell cultures and animals [72–76] and many studies observed an up-regulation of Trxr-1 and Trx-1 triggered by resveratrol [77, 78] but no studies investigated PTS effect on Trxr-1. Moreover, Ho and Trxr-1 are classified as vitagenes, a group of genes which are strictly involved in preserving cellular homeostasis during stressful conditions [79]. The vitagene family is composed of the heat shock proteins (Hsp) Hsp32 (also known as HO-1), Hsp70, sirtuins and by the thioredoxin system [70, 80–82]. In agreement with the data on the protection against oxidative stress, PTS up-regulated Ho and Trxr-1 only in male flies. Our hypothesis is that PTS is more effective in inducing the antioxidant system in males in respect to females because the female endogenous antioxidant system is already highly expressed, and maybe PTS is not able to trigger a further increase.

Many studies have associated an anti-inflammatory activity to PTS. It has been described that PTS inhibited mitogen-activated protein kinase (MAPK) phosphorylation and the production of pro-inflammatory cytokines (Interleukin-6 and TNF-*α*) in mouse microglial cells activated by lipopolysaccharides [83]. Moreover, PTS showed inhibitory effect on inflammatory responses following the interaction of 3 T3-L1 adipocytes with RAW 264.7 macrophages [84]. Our data demonstrated that PTS supplementation reduced the expression of two pro-inflammatory cytokines, in particular dome, that has similarities with the mammalian IL-6 receptor family [9] and egr, the fly orthologue of TNF*α* [51], in female flies, but had no effect in male flies.

We performed proteomic analyses of male and female flies supplemented for 15 days with PTS to better characterized the mechanisms behind the higher protection of PTS against oxidative stress in males in respect to female flies. Unlike what we would have expected, the proteomics data did not provide further information useful for understanding the different effect of PTS supplementation on oxidative stress. Nevertheless, several interesting aspects emerged. The results showed that PTS modulates a higher number of proteins in male in respect to female flies (113 and 9, respectively). Among the identified proteins, only one is in common between the two sexes: vacuolar H[+] ATPase 68 kDa subunit 2 (Vha68-2).The strong down-regulation of Vha68-2 by PTS is comparable in the two sexes. This protein is part of the vacuolar proton-translocating ATPase (V-ATPase) that is organized into 2 subcomplexes, namely, the ATP-catalyzing domain (V1) and a proton-translocation domain (V0) and Vha68-2 belongs to V0 domain. V-ATPase is a key regulator of organelle acidification in eukaryotic cells [85, 86] and has been demonstrated to modulate several cellular processes, like membrane trafficking and protein degradation [86, 87]. In *D. melanogaster* the role of Vha68-2 has been marginally investigated in only one study related to the function of *Drosophila* salivary glands during the early- to mid-prepupal period [88]. However, a role of V-ATPase in longevity has been demonstrated in *C. elegans* where the RNAi downregulation of 2 different subunits of the V-ATPase, vha-3 and vha-12 belonging to V0 and V1, respectively, increased lifespan. The authors suggested that this lifespan extension might be due to Ce.TOR inhibition, because V-ATPase is required for the spatial regulation and subsequent activation of TORC1 [89]. On the other hand, only in male flies, vacuolar proton pump subunit B (Vha55), that corresponds to vha-21 in *C. elegans* [90], is induced by PTS supplementation. These observations suggest that Vha68-2 downregulation by PTS could be involved in the observed increase in both male and female lifespan. Further studies should be carried out to better investigate this point and to verify how this protein is modulated in older flies.

Among the proteins modulated by PTS in female flies, enolase and pyruvate dehydrogenase are particularly interesting as are enzymes related to glucose metabolism, the first one is a glycolytic enzyme, meanwhile the second one transforms the product of glycolysis, pyruvate, in acetyl CoA. Both are strongly downregulated, and this is in agreement with the observations that caloric restriction is characterized by a decreased glucose metabolism [91, 92]. Moreover, Schriner et al. [91], studying the mechanisms behind *Rhodiola rosea* ability to extend life span in *Drosophila*, evidenced that *R. rosea* significantly reduced the expression of enolase in female and not in male flies and associated this effect with lifespan increase. This is supported by our data as we observed the downregulation of this enzyme only in female flies that showed a higher mean lifespan in respect to male flies. The limitation of our results is that they were obtained analyzing relatively young flies (15 days old) and as underlined previously, to draw more reliable conclusions, further studies should be carried out to investigate the proteome of older flies.

Among the proteins modulated by PTS in male flies, malate dehydrogenase 2 (mdh2) shows the highest upregulation (fold >3). Mdh2 is closely related to mitochondrial malate dehydrogenases from multiple animal species, including mouse Mdh2 and yeast Mdh1 and localizes to mitochondria *in vivo* [93]. Its main role is to transform malate to oxalacetate in the tricarboxylic acid cycle and is critical for cellular energy production. This is supported by the observation of Wang et al. [93] that observed significantly lower levels of ATP in Mdh2 *Drosophila* mutants and an accumulation of late-stage citric acid cycle intermediates. Of note, this protein is strongly up-regulated in male and not in female flies and this suggests that this up-regulation triggered by PTS could be linked to the presence of specific male hormones. In general, PTS induces different proteins related to metabolic pathways, among them pyruvate carboxylase and kinase, succinate-semialdehyde dehydrogenase, 3-hydroxy-3-methylglutaryl coenzyme A synthase, and ATP-dependent 6-phosphofructokinase. On the other hand, PTS downregulates few proteins, among them Vha68-2, previously discussed, Annexin B10 (AnxB10), electron transfer flavoprotein subunit beta (ETFB), 14-3-3 protein zeta (14-3-3zeta), and translationally-controlled tumor protein homolog (Tctp). Interestingly, in *D. melanogaster* it has been demonstrated that 14-3-3 genes show strong genetic interaction with Tctp [94], a protein involved in Tor signalling [94–97]. In particular, in *Drosophila*, Tctp is essential for organ growth by promoting Rheb function for Tor signalling as a guanine nucleotide exchange factor [95]. Moreover, Tctp is over-expressed in cancer cells, and its downregulation induces the reversion of tumour phenotypes [98–100]. From this point of view, the reduction of Tcpt triggered by PTS supplementation in male flies could have a role in counteracting tumorigenesis. Further studies should be carried out to better clarify this interesting aspect.

## 5. Conclusions

In conclusion, our data demonstrate for the first time that PTS increases average lifespan of both male and female flies. Interestingly, the mechanisms behind this effect are different in the two sexes. Protein related to longevity are modulated with different time patterns in the two sexes, moreover PTS was able to increase the expression of two genes (Ho and Trxr-1) involved in the antioxidant defense only in male flies and reduces pro-inflammatory proteins only in female flies. Proteomic analysis suggests a potential involvement of Vha68-2 in the observed increase of lifespan, a reduction of glucose metabolism in female flies and an induction of different metabolic pathways in male flies together with the downregulation of 14-3 3zeta and Tctp suggesting a potential role of PTS in reducing tumorigenesis. These data show that males and females respond differently to treatments, reinforcing the emerging idea that studies on drugs and nutraceuticals should be conducted separately in the two sexes to give more reliable answers. Moreover, these results stressed on the importance of creating personalized treatment, that take into consideration the genetic and biological diversity that arise among different individuals and among the two sexes.

## Figures and Tables

**Table 1:** List of primers for real-time PCR.

Gene	5'-Forward-3'	5'-Reverse-3'
Sir2	CATTATGCCGCATTTCGCCA	GAAGGTGTTCACTGAGGCCA
Foxo	AGGCTGACCCACACAGATAAC	GGCTCCACAAAGTTTTCGGG
Notch	CGCTTCCTGCACAAGTGTC	GCGCAGTAGGTTTTGCCATT
Ho	ATGTCAGCGAGCGAAGAAACA	TGGCTTTACGCAACTCCTTTG
Trxr-1	TGGATCTGCGCGACAAGAAAG	GAAGGTCTGGGCGGTGATTG
Dome	GGCAGCTTCTATGTCTACTC	GTTGGACTCCACCTTGATG
Egr	GAAATCACACAGAGCTTCAG	AAGAAGAGATTCACCTTTGC
RPL32	GCCCACCGGATTCAAGAAGT	CTTGCGCTTCTTGGAGGAGA

**Table 2:** List of differentially expressed proteins identified by nLC-ESI-MS/MS.

Spot n°	Accession (UniProtKB)	Gene symbol	Protein name	Cov [%]	PSMs	Unique peptides	MW [kDa]	Calc. pI	Score	Ratio (PTS/CTR)	p value
	**FEMALES**										
818	Q24253	AP-1-2beta	AP complex subunit beta	46	68	45	101.1	5.11	227	1.3	0.00087
1210	Q9VA70	CDase	Neutral ceramidase	12	6	6	78.2	6.6	19	0.45	0.00055
1351	Q9VWV6	Tsf1	Transferrin	63	71	42	71.8	7.06	233	1.5	0.00094
1450	A4V0N4	Vha68-2	H(+)-transporting two-sector ATPase	49	38	18	68.3	5.34	142	0.63	0.00074
1762	Q7KW39	CG17896	Probable methylmalonate-semialdehyde dehydrogenase [acylating], mitochondrial	34	21	15	55.9	8.35	85	0.83	0.001
1803	Q24560	betaTub56D	Tubulin beta-1 chain	69	191	11	50.1	4.86	627	1.2	0.00039
2070	M9NH07	Up	Upheld, isoform N	41	35	2	47.2	4.96	102	0.7	0.00068
2080	E1JHR5	Eno	Enolase	42	19	15	46.6	6.55	73	0.48	0.000096
2731	Q7K5K3	Pdhb	Pyruvate dehydrogenase E1 component subunit beta	34	17	12	39.3	7.8	60	0.56	0.00012
	**MALES**										
649	Q7KN97	PCB	Pyruvate carboxylase	61	92	65	130.8	6.81	312	1.5	0.001
650	Q7KN97	PCB	Pyruvate carboxylase	62	116	70	130.8	6.81	385	1.67	0.005
788	H9XVM8	Dmel\CG1674	Uncharacterized protein, isoform K	37	33	7	96.2	6.7	107	1.62	0.00039
947	Q7KN62	TER94	Transitional endoplasmic reticulum ATPase TER94	72	135	5	88.8	5.35	455	1.58	0.002
1047	Q9VHP0	Bel	ATP-dependent RNA helicase bel	18	13	12	85	7.53	41	1.70	0.004
1064	Q9VNW6	P5CS	Delta-1-pyrroline-5-carboxylate synthase	48	39	31	84	6.73	135	1.56	0.013
1073	Q9VNW6	P5CS	Delta-1-pyrroline-5-carboxylate synthase	54	64	35	84	6.73	230	1.57	0.004
1177	P52034	Pfk	ATP-dependent 6-phosphofructokinase	58	64	42	86.6	6.83	232	1.91	0.002
	Q9VNV3	Ddx1	ATP-dependent RNA helicase Ddx1	11	6	6	80.8	6.87	16		
1450	A4V0N4	Vha68-2	H(+)-transporting two-sector ATPase	49	38	18	68.3	5.34	142	0.68	0.007
1483	Q07327	Rop	Protein ROP	52	50	31	67.8	6.7	193	1.78	0.006
1655	O62619	PyK	Pyruvate kinase	55	31	23	57.4	7.44	115	1.97	0.005
1837	E1JIJ5	Vha55	Vacuolar proton pump subunit B	54	92	25	54.5	5.4	318	1.65	0.014
1845	Q9VBP6	Ssadh	Succinate-semialdehyde dehydrogenase	50	26	22	54.9	8.21	85	1.55	0.00073
	Q9W058	SCOT	Succinyl-CoA:3-ketoacid-coenzyme A transferase, mitochondrial	43	24	16	54.9	8.37	82		
1876	Q9VEP6	AdSL	Adenyluccinate lyase	43	30	20	53.8	7.49	103	1.5	0.013
1943	Q7K4Q9	Hmgs	3-hydroxy-3-methylglutaryl coenzyme A synthase	54	30	19	51.1	6.32	112	1.52	0.022
2389	Q9VSA3	CG12262	Probable medium-chain specific acyl-CoA dehydrogenase, mitochondrial	47	64	24	45.8	7.94	207	1.51	0.015
2600	M9PF46	Prps	Ribe-phphate diphphokinase	58	34	22	40.9	7.91	120	1.57	0.028
2812	P22465	AnxB10	Annexin B10	29	7	7	35.7	4.74	24	0.67	0.018
2894	Q9VEB1	Mdh2	Malate dehydrogenase	54	71	23	35.3	9.11	237	3.46	0.00060
3049	Q9VM18	21430192	Trehalose 6-phosphate phosphatase	33	10	9	31.2	7.12	29	1.5	0.004
	Q7KN94	Wal	Electron transfer flavoprotein subunit alpha	44	46	13	34.2	8.32	148		
3239	Q0KHZ6	ETFB	Electron transfer flavoprotein subunit beta	79	51	21	27.2	8.05	177	0.73	0.023
	Q7JR58	Echs1	Enoyl-CoA hydratase, short chain 1, isoform A	39	14	10	31.6	8.63	47		
3301	P29310	14-3-3zeta	14-3-3 protein zeta	41	10	9	28.2	4.88	30	0.42	0.015
3409	P00334	Adh	Alcohol dehydrogenase	68	66	12	27.7	7.96	234	1.60	0.013
3731	Q9VGS2	Tctp	Translationally-controlled tumor protein homolog	80	43	17	19.6	4.81	140	0.71	0.004

Cov [%] = coverage [%], the percentage of the sequence covered by identifications (PSMs only) from the included searches; PSMs = the total number of peptide spectrum matches identified from all included searches; Unique Peptides: the total number of distinct peptide sequences unique to the protein group; MW [kDa]: the molecular weight of the protein; calc. pI: the isoelectric point calculated for the protein; Score = Score Sequest HT, the protein score which is calculated by summing the individual scores of each peptide. The higher this score, the higher the individual scores of the peptides, and thus the better the identification. Sequest HT is the name of the employed search engine.

## Data Availability

The data used to support the findings of this study are available from the corresponding author upon request.
